# Facilitating Uniform Large-Scale MoS_2_, WS_2_ Monolayers, and Their Heterostructures through van der Waals Epitaxy

**DOI:** 10.1021/acsami.2c12174

**Published:** 2022-09-09

**Authors:** Chung-Che Huang, He Wang, Yameng Cao, Ed Weatherby, Filipe Richheimer, Sebastian Wood, Shan Jiang, Daqing Wei, Yongkang Dong, Xiaosong Lu, Pengfei Wang, Tomas Polcar, Daniel W. Hewak

**Affiliations:** † Optoelectronics Research Centre, University of Southampton, Southampton SO17 1BJ, United Kingdom; ‡ nCAT, University of Southampton, Southampton SO17 1BJ, United Kingdom; § National Physical Laboratory, Teddington, TW11 0LW, United Kingdom; ∥ School of Materials Science and Engineering, Harbin Institute of Technology, 150001 Harbin, China; ⊥ National Key Laboratory of Science and Technology on Tunable Laser, Harbin Institute of Technology, 150001 Harbin, China; # School of Physics and Electronic Engineering, Jiangsu Normal University, 221116 Xuzhou, China; ∇ Key Laboratory of In-Fiber Integrated Optics of Ministry of Education, College of Science, Harbin Engineering University, 150001 Harbin, China

**Keywords:** MoS_2_, WS_2_, heterostructures, van der Waals epitaxy, transition-metal dichalcogenides, nanoelectronics, nanophotonics

## Abstract

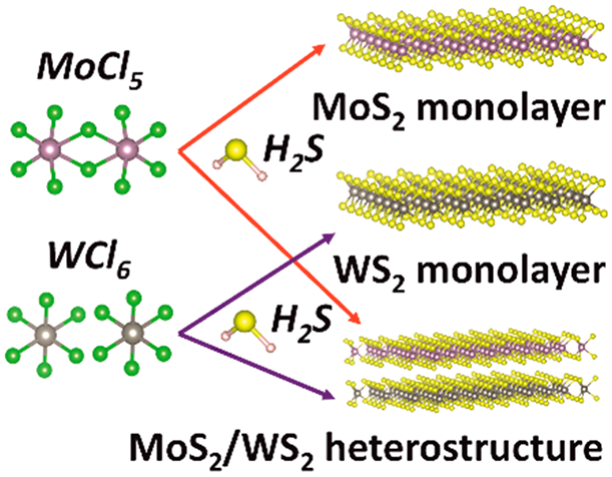

The fabrication process for the uniform large-scale MoS_2_, WS_2_ transition-metal dichalcogenides (TMDCs) monolayers, and their heterostructures has been developed by van der Waals epitaxy (VdWE) through the reaction of MoCl_5_ or WCl_6_ precursors and the reactive gas H_2_S to form MoS_2_ or WS_2_ monolayers, respectively. The heterostructures of MoS_2_/WS_2_ or WS_2_/MoS_2_ can be easily achieved by changing the precursor from WCl_6_ to MoCl_5_ once the WS_2_ monolayer has been fabricated or switching the precursor from MoCl_5_ to WCl_6_ after the MoS_2_ monolayer has been deposited on the substrate. These VdWE-grown MoS_2_, WS_2_ monolayers, and their heterostructures have been successfully deposited on Si wafers with 300 nm SiO_2_ coating (300 nm SiO_2_/Si), quartz glass, fused silica, and sapphire substrates using the protocol that we have developed. We have characterized these TMDCs materials with a range of tools/techniques including scanning electron microscopy (SEM), X-ray photoelectron spectroscopy (XPS), micro-Raman analysis, photoluminescence (PL), atomic force microscopy (AFM), transmission electron microscopy (TEM), energy-dispersive X-ray spectroscopy (EDX), and selected-area electron diffraction (SAED). The band alignment and large-scale uniformity of MoS_2_/WS_2_ heterostructures have also been evaluated with PL spectroscopy. This process and resulting large-scale MoS_2_, WS_2_ monolayers, and their heterostructures have demonstrated promising solutions for the applications in next-generation nanoelectronics, nanophotonics, and quantum technology.

## 1 Introduction

Transition-metal dichalcogenides (TMDCs) such as MoS_2_, MoSe_2_, WS_2_, and WSe_2_ are two-dimensional (2D) van der Waals (VdW) layered materials. Unlike graphene, TMDCs are semiconductors that could offer, in particular, bandgap engineering properties through both their chemical compositions and their number of layers.^1,2^ The applications for using TMDCs are very promising in the area of transistors,^1^ light-emitting diodes,^3,4^ photodetectors,^5^ sensing^6,7^ and memory devices,^8^ as well as the potential substitution for Si in conventional electronics^9^ and of organic semiconductors in wearable and flexible systems.^10^

The current fabrication processes for these emerging TMDCs include exfoliation,^1,11^ hydrothermal process,^12^ physical vapor deposition,^13^ transition-metal oxide sulfurization,^14^ electrochemical deposition,^15^ thermolysis of transition-metal chalcogenide compounds^16,17^ and chemical vapor deposition (CVD).^18−20^ The majority of TMDCs fabricated by these techniques are in the form of flakes with the sizes in the range of a few hundred square micrometers in area. However, the challenge for large-scale fabrication of TMDCs is to provide a reliable complementary metal-oxide-semiconductor (CMOS)-compatible process for the integration of 2D TMDCs on a desired wafer-scale substrate.^2,21^

We have been working on the synthesis of chalcogenide materials using vapor phase deposition processes^22−27^ such as CVD, atomic layer deposition (ALD), and van der Waals epitaxy (VdWE). Apart from offering conformal coating and stoichiometric control of thin film compositions, these processes are scalable and compatible with a range of substrates. In particular, VdWE has been demonstrated to perform the epitaxy of layered TMDCs on the substrates even with lattice constants mismatch.^28−30^ In this paper, we have developed the fabrication process for the uniform large-scale MoS_2_, WS_2_ TMDCs monolayers and their heterostructures by VdWE through the reaction of MoCl_5_ or WCl_6_ precursors and the reactive gas H_2_S to form MoS_2_ or WS_2_ monolayers, respectively. The heterostructures can easily be achieved by changing the precursor from WCl_6_ to MoCl_5_ once the initial WS_2_ monolayer is fabricated or switching the precursor from MoCl_5_ to WCl_6_ after MoS_2_ monolayer has been deposited on the substrate.

## 2 Experimental Setup

**Figure 1:**
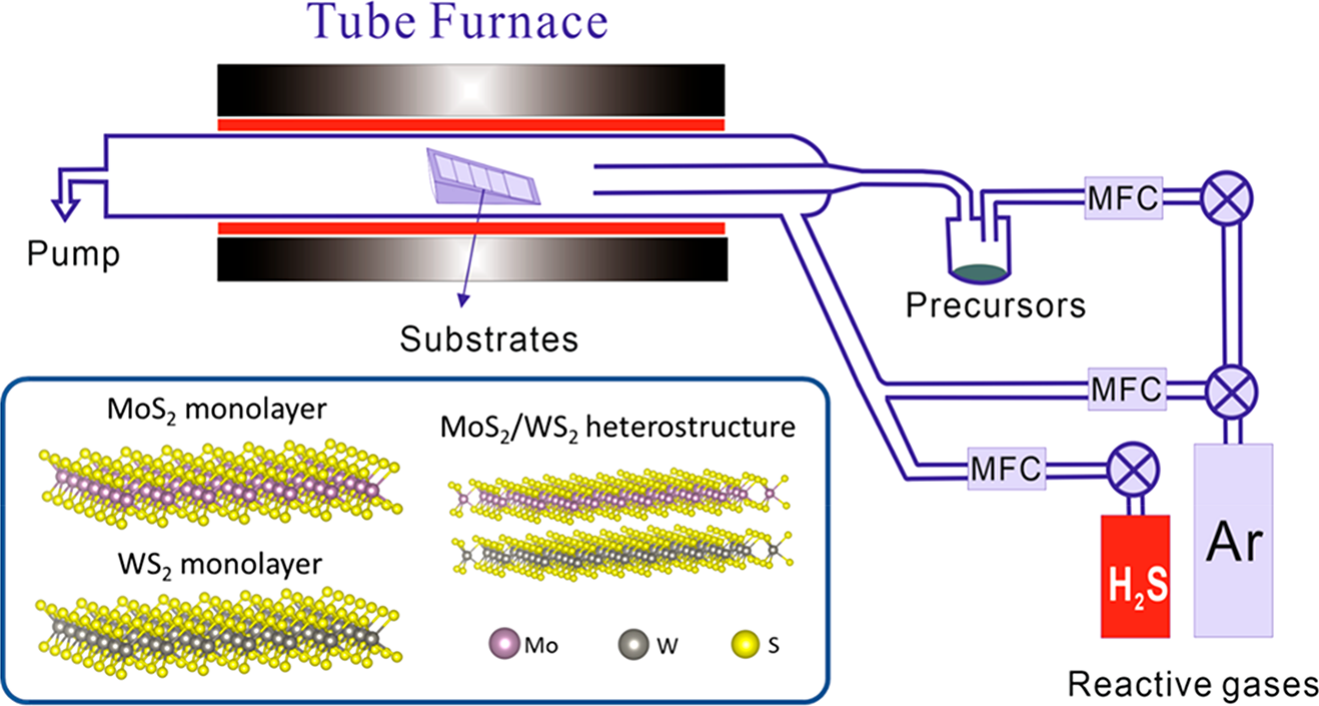
Schematic van der Waals epitaxy (VdWE) apparatus for the fabrication of MoS_2_, WS_2_, and their heterostructures.

The VdWE apparatus we developed is shown schematically in Figure 1. The precursors—MoCl_5_ (99.6% pure from Alfa Aesar) and WCl_6_ (99.9% pure from Sigma-Aldrich)—were kept in bubblers inside the dry N_2_ purged glovebox. The MoCl_5_/WCl_6_ vapors were delivered by high-purity argon gases through the mass flow controllers (MFCs) to the VdWE apparatus with the flow rate of 300 standard cubic centimeters per minute (sccm). The system equipped with a bespoke furnace with three heating zones, individually controlled by proportional integral derivative (PID) controllers, with maximum temperature of 1200 °C and temperature uniformity of ±3 °C can be achieved over a length of 450 mm to facilitate the uniform large-scale deposition of TMDC monolayers. The reactive gases were H_2_S mixed with another argon gas through individual MFCs with the flow rates of 50 and 300 sccm, respectively. All the gases were purified by passing through the individual point of use purifiers (SAES MicroTorr) and the moisture level of all gases were monitored by the dewpoint sensors (Michell Instrument Pura pure gas trace moisture transmitters) before entering the VdWE reactor. The typical moisture readings of the Ar and H_2_S/Ar mixture were −99.6 °C dp (∼7 ppb) and −90.2 °C dp (∼42 ppb), respectively. The process was set at 30 mbar using a pump (Vacuubrand MV 10C NT Vario) with a pressure controller for the entire deposition. With this VdWE apparatus, uniform large-scale TMDC monolayers have been successfully deposited on various substrates, including 300 nm SiO_2_/Si, quartz glass, fused silica, or *c*-plane sapphire. The sizes of the substrates were typically 25 mm × 25 mm, however up to a 40 mm × 100 mm substrate can be loaded into the VdWE apparatus, which consists of a 50 mm O.D. × 1000 mm long quartz reaction tube. The substrates were cleaned with acetone using an ultrasonic bath at 50 °C for 10 min, then rinsed with isopropanol and deionized water and subsequently subjected to blow drying with N_2_ gas. The temperatures for the growth of MoS_2_ and WS_2_ monolayers were set at 850 and 900 °C, respectively. The reactive H_2_S gas and MoCl_5_/WCl_6_ precursors were introduced to the VdWE system once the furnace reached the set temperature. MoS_2_/WS_2_ were formed after the MoCl_5_/WCl_6_ precursors met with H_2_S gas after the injection tube inside the quartz reaction tube. With sufficient amount of MoCl_5_/WCl_6_ precursors flux, MoS_2_/WS_2_ monolayers can be uniformly deposited on the substrates and the resulting MoS_2_/WS_2_ monolayers have a tendency to be polycrystalline, because of the high flux of precursors. Although the substrates might affect the deposition of TMDCs, we did not see significant differences in the quality of the MoS_2_, WS_2_ monolayers, and their heterostructures on the substrates we used. This is probably due to the VdWE process can overcome the mismatch of substrate lattice constants. To achieve uniform MoS_2_ and WS_2_ monolayers, a deposition time of 4 and 5 min was required for the MoS_2_ and WS_2_ monolayers, respectively.

## 3 Results and Discussion

**Figure 2:**
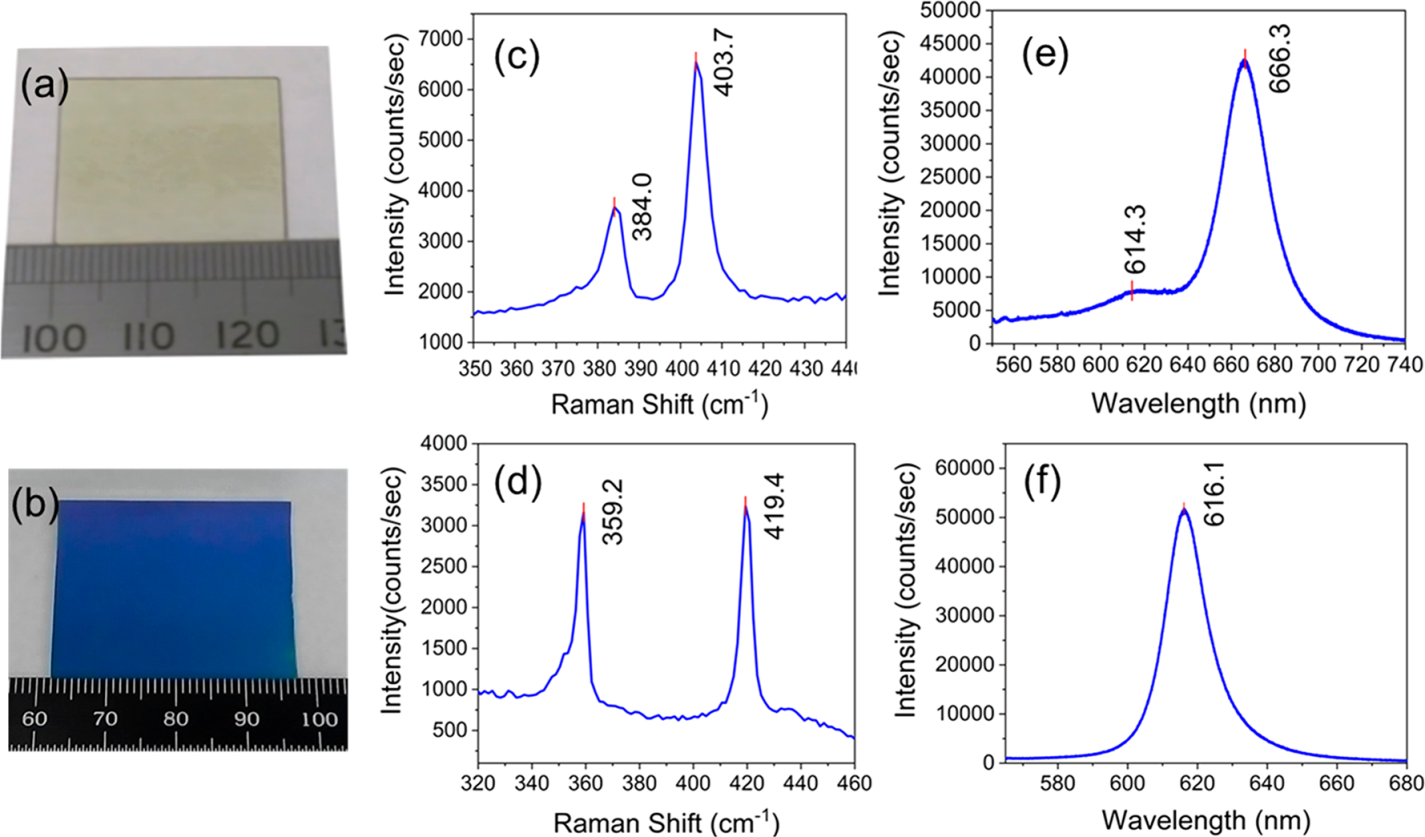
(a) Photograph of VdWE-grown MoS_2_ monolayer on a quartz glass substrate, (b) photograph of WS_2_ monolayer on a 300 nm SiO_2_/Si substrate, (c) Raman spectrum of VdWE-grown MoS_2_ monolayer on quartz glass (with 532 nm excitation laser), (d) Raman spectrum of VdWE-grown WS_2_ monolayer on 300 nm SiO_2_/Si substrate (with 473 nm excitation laser), (e) photoluminescence (PL) spectrum of VdWE-grown MoS_2_ monolayer on quartz glass (with 532 nm excitation laser), and (f) PL spectrum of VdWE-grown WS_2_ monolayer on a 300 nm SiO_2_/Si substrate (with 532 nm excitation laser).

We have achieved large area MoS_2_ and WS_2_ monolayers as shown in Figure 2a on quartz glass and in Figure 2b on 300 nm SiO_2_/Si substrates, respectively. These results have demonstrated that wafer scale deposition of MoS_2_ and WS_2_ monolayers is feasible through a modification of the VdWE system with a larger reaction chamber.

Raman spectroscopy was performed for the initial study of the quality of the VdWE-grown MoS_2_ and WS_2_ monolayers on quartz glass and 300 nm SiO_2_/Si substrates, using a Renishaw Ramascope. MoS_2_ monolayer and WS_2_ monolayer samples were excited using 532 and 473 nm excitation lasers, and the Raman shift spectra for MoS_2_ and WS_2_ are shown in Figures 2c and 2d, respectively. As shown in Figure 2c, two MoS_2_ Raman peaks, E_2g_^1^ in-plane phonon mode and A_1g_ out-of-plane phonon mode were revealed at 384.0 and 403.7 cm^–1^, respectively. The number of MoS_2_ layers can be evaluated by the energy difference between these two Raman peaks (Δ).^31^ From Figure 2c, the Δ value is 19.7 cm^–1^ for the VdWE-grown MoS_2_ monolayer, which is similar to the reported literature.^31^ On the other hand, in order to reduce the second-order 2LA phonon mode in the WS_2_ Raman measurement,^32^ a 473 nm laser was used to reveal two WS_2_ Raman peaks, E_2g_^1^ and A_1g_ at 359.2 and 419.4 cm^–1^, respectively. Again, the Δ value can be also used to evaluate the number of WS_2_ layers.^32^ From Figure 2d, the Δ value is 60.2 cm^–1^ for the VdWE-grown WS_2_ monolayer, which also matches with the literature.^32^

The photoluminescence (PL) spectroscopy from the VdWE-grown MoS_2_ monolayer on quartz glass and WS_2_ monolayer on 300 nm SiO_2_/Si substrates were studied using the same Raman microscope. Two excitonic peaks A and B, at 666.3 nm (1.86 eV) and 614.3 nm (2.02 eV), respectively, were found in the PL spectrum of VdWE-grown MoS_2_ monolayer on a quartz glass substrate, as shown in Figure 2e. These results are similar to the reported literature.^33^ On the other hand, the PL spectrum of VdWE-grown WS_2_ monolayer on 300 nm SiO_2_/Si substrate confirmed the direct band emission at 616.1 nm (2.01 eV), as shown in Figure 2f. Again, this result agrees with the literature reports.^34^

**Figure 3:**
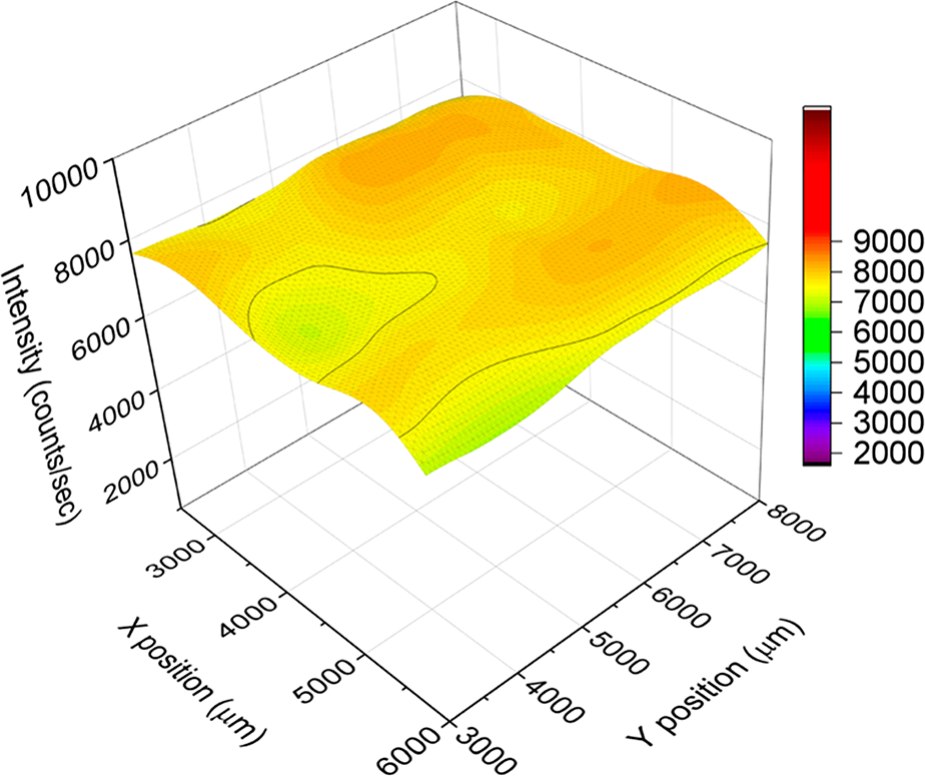
Photoluminescence spectra mapping at 2.01 eV of VdWE-grown WS_2_ monolayer on a 300 nm SiO_2_/Si substrate.

Furthermore, the PL spectra mapping was performed to study the uniformity of large-scale VdWE-grown WS_2_ monolayer on a 300 nm SiO_2_/Si substrate. The map of the PL emission at 2.01 eV shown in Figure 3 reveals very good uniformity of the WS_2_ monolayer over an area of 35 mm × 50 mm. This has been achieved by our in-house-built apparatus, and this process could be scalable for even large wafer-scale processes if a larger reactor is available.

**Figure 4:**
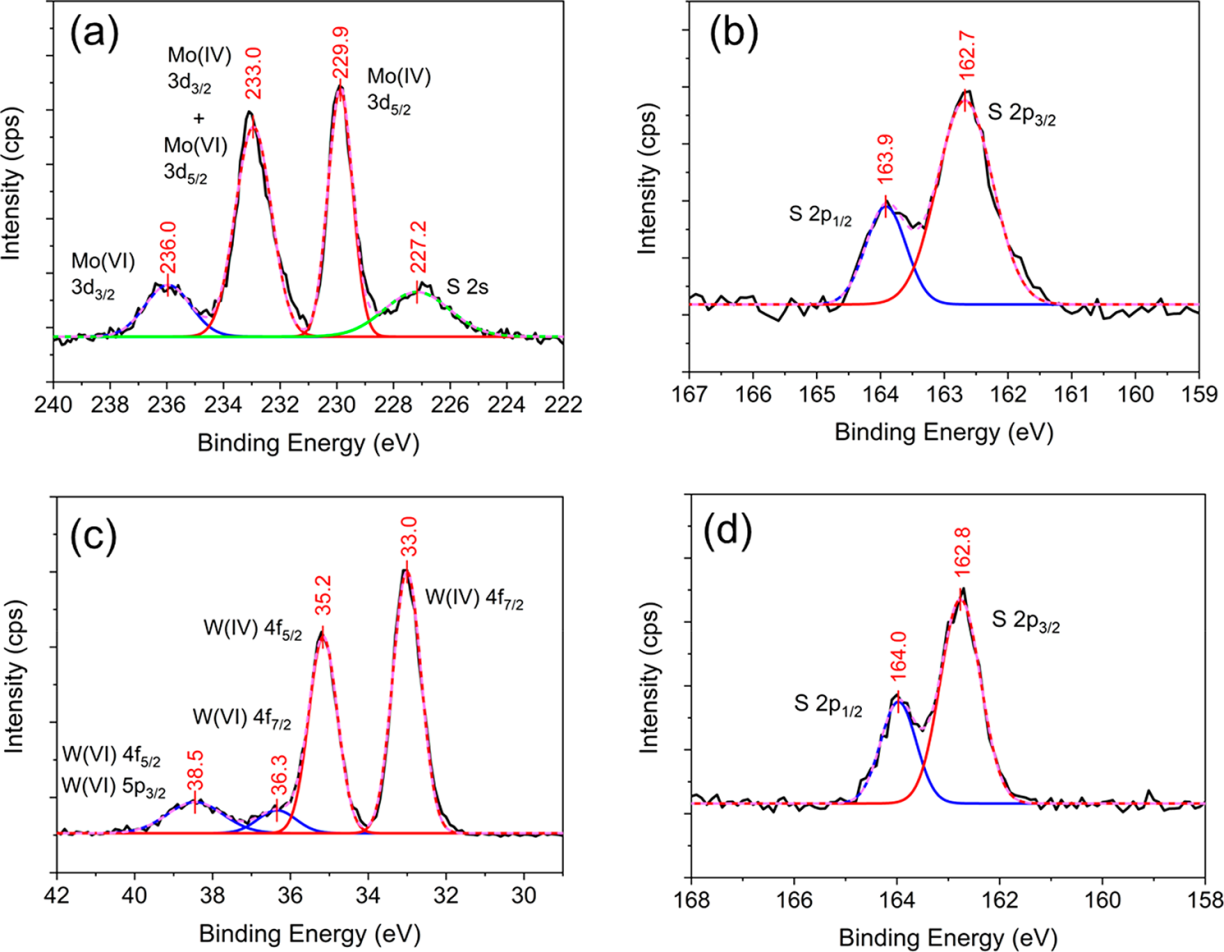
XPS measurements of VdWE-grown MoS_2_ and WS_2_ monolayers (a) Mo 3d scan of MoS_2_ monolayer, (b) S 2p scan of MoS_2_ monolayer, (c) W 4f scan of WS_2_ monolayer, and (d) S 2p scan of WS_2_ monolayer on a 300 nm SiO_2_/Si substrate.

X-ray photoelectron spectroscopy (XPS) was performed to study the compositions of these VdWE-grown MoS_2_ and WS_2_ monolayers using a Thermo Scientific Theta Probe XPS System. For the MoS_2_ monolayer, two core levels, Mo 3d and S 2p, have been investigated. As shown in Figure 4a, two MoS_2_ peaks, Mo(IV) 3d_3/2_ and Mo(IV) 3d_5/2_, were found at 233.0 and 229.9 eV, respectively. In the same spectrum, S 2s peak was observed at 227.2 eV and a peak at 236.0 eV was assigned to Mo(VI) 3d_3/2_, indicating a small amount of oxidation, which resulted from the sample being exposed to the ambient environment. Note that a Mo(VI) 3d_5/2_ peak overlaps with Mo(IV) 3d_3/2_ at 233.0 eV. For the MoS_2_ S 2p core level, two peaks labeled in Figure 4b as S 2p_1/2_ and S 2p_3/2_ corresponding to MoS_2_ were found at 163.9 and 162.7 eV, respectively. In addition, using a semiquantitative method to investigate the ratio of elements, the atomic ratio of S/Mo was determined to be ∼1.93 with a slight S deficiency. These results are consistent with the literature.^35^ On the other hand, for the WS_2_ monolayer, two core levels have been studied: W 4f and S 2p. As shown in Figure 4c, two WS_2_ peaks, W(IV) 4f_5/2_ and W(IV) 4f_7/2_, were found at 35.2 and 33.0 eV, respectively, and in the same spectrum, two peaks at 38.5 and 36.3 eV were assigned to W(VI) 4f_5/2_ and W(VI) 4f_7/2_, which again indicate a small amount of oxidation. Also, note that the W(VI) 4f_5/2_ peak overlaps with W(VI) 5p_3/2_ at 38.5 eV. For the WS_2_ S 2p core level, two peaks labeled in Figure 4d as S 2p_1/2_ and S 2p_3/2_, corresponding to WS_2_, were found at 164.0 and 162.8 eV, respectively. In addition, the atomic ratio of S/W was found to be ∼1.96, with a slight S deficiency. These results also agree very well with the literature.^36^

**Figure 5:**
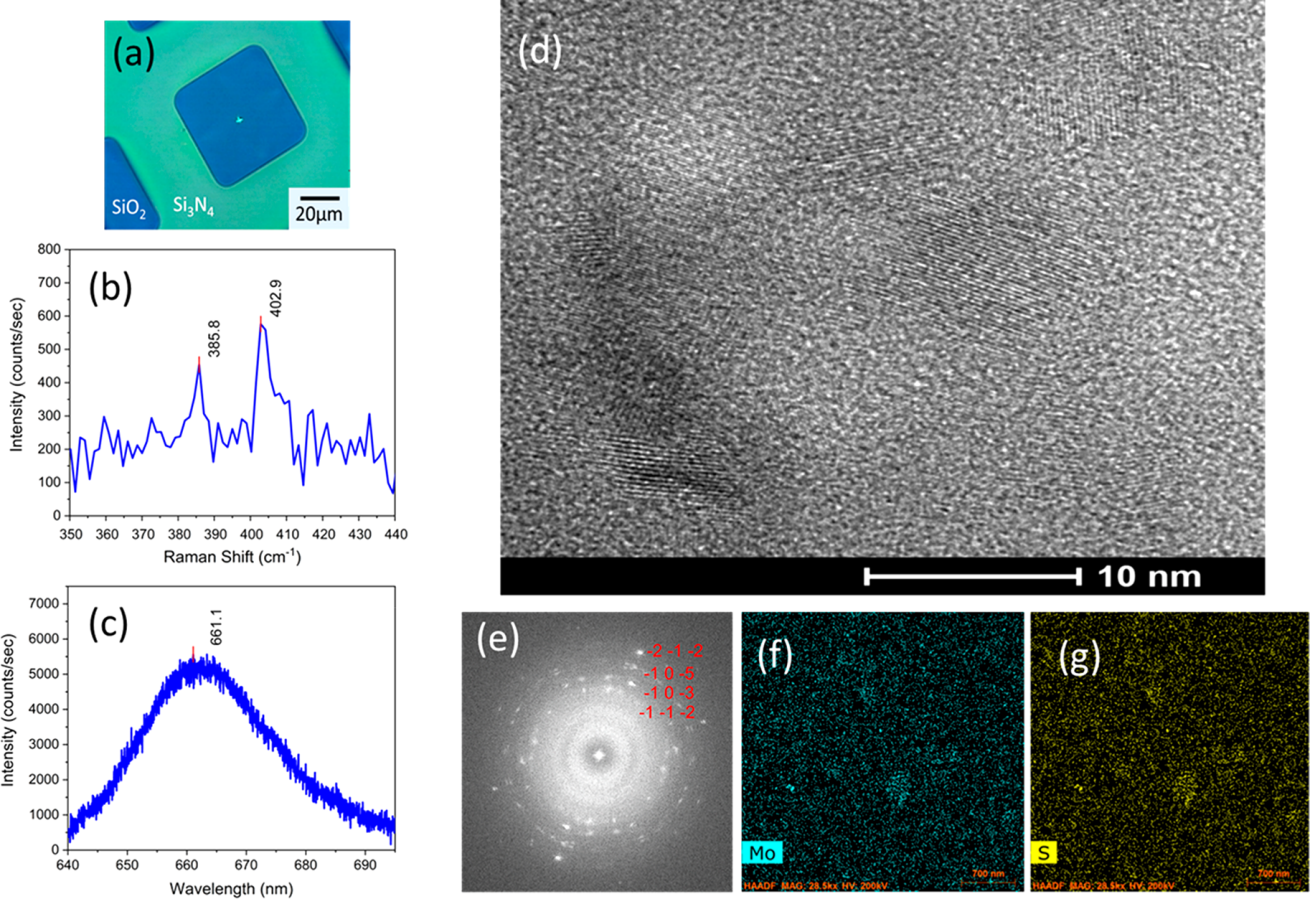
TEM measurements of VdWE-grown MoS_2_ monolayer on a 40 nm SiO_2_ membrane/Si_3_N_4_/Si TEM grid: (a) photograph of the sample with a 532 nm laser spot, (b) Raman spectrum of the VdWE-grown MoS_2_ monolayer sample, (c) PL spectrum of the VdWE-grown MoS_2_ monolayer sample, (d) TEM image of the VdWE-grown MoS_2_ monolayer sample, (e) selected-area electron diffraction (SAED) pattern of the VdWE-grown MoS_2_ monolayer sample, (f) energy-dispersive X-ray spectroscopy (EDX) mapping of Mo atom on the selected area of MoS_2_ monolayer sample, and (g) EDX mapping of S atom on the selected area of the MoS_2_ monolayer sample.

In order to evaluate the crystalline structures of these VdWE-grown MoS_2_ or WS_2_ monolayers, commercially available 40 nm SiO_2_ membranes TEM grids with 200-nm-thick Si_3_N_4_ supporting frames (PELCO Silicon Dioxide Support Films for TEM) were used to directly deposit these MoS_2_ and WS_2_ monolayers on this type of TEM grid. The optical image of as-deposited MoS_2_ monolayer on TEM grid is shown in Figure 5a with a 532 nm laser spot on the center of the MoS_2_/40 nm SiO_2_ membrane. The Raman spectrum of the MoS_2_ monolayer/40 nm SiO_2_ sample is shown in Figure 5b. Again, two characteristic MoS_2_ Raman peaks, E_2g_^1^ and A_1g_ modes were found at 385.8 and 402.9 cm^–1^, respectively, with a Δ value of 17.1 cm^–1^ for the VdWE-grown MoS_2_ monolayer on a 40 nm SiO_2_ membrane TEM grid. Note that the Δ value appears to be less than that typically reported for the MoS_2_ monolayer, because of the weak Raman signal from the sample, which increased the experimental uncertainty. In addition, the smaller Δ value could be also due to softening of the A_1g_ mode. The E_2g_^1^ mode is insensitive to substrates but the A_1g_ mode is sensitive to charge density.^37^ Despite these issues, however, the monolayer nature has been revealed. In the PL spectrum, shown in Figure 5c, only the A excitonic peak at 661.1 nm (∼1.88 eV) was found for this sample on a 40 nm SiO_2_ TEM membrane, whereas the B exciton could be only weakly detected. The sample was inspected using scanning tunnelling electron microscopy with a high-angle-annular-dark-field (HAADF-STEM), using a FEI Talos F200x system (USA), operating at 200 kV and equipped with an energy-dispersive X-ray spectrometer (EDX) system. The TEM image shown in Figure 5d has revealed the polycrystalline nature of the VdWE-grown MoS_2_ monolayer on a 40 nm SiO_2_ TEM membrane, and the grain sizes are ∼10 nm. The selected-area electron diffraction (SAED) patterns shown in Figure 5e also confirmed the polycrystalline structures of this MoS_2_ monolayer. The elemental mapping was performed in the STEM-EDX mode. As shown in Figures 5f and 5g, the Mo and S, respectively, were quite uniform over the measured area.

**Figure 6:**
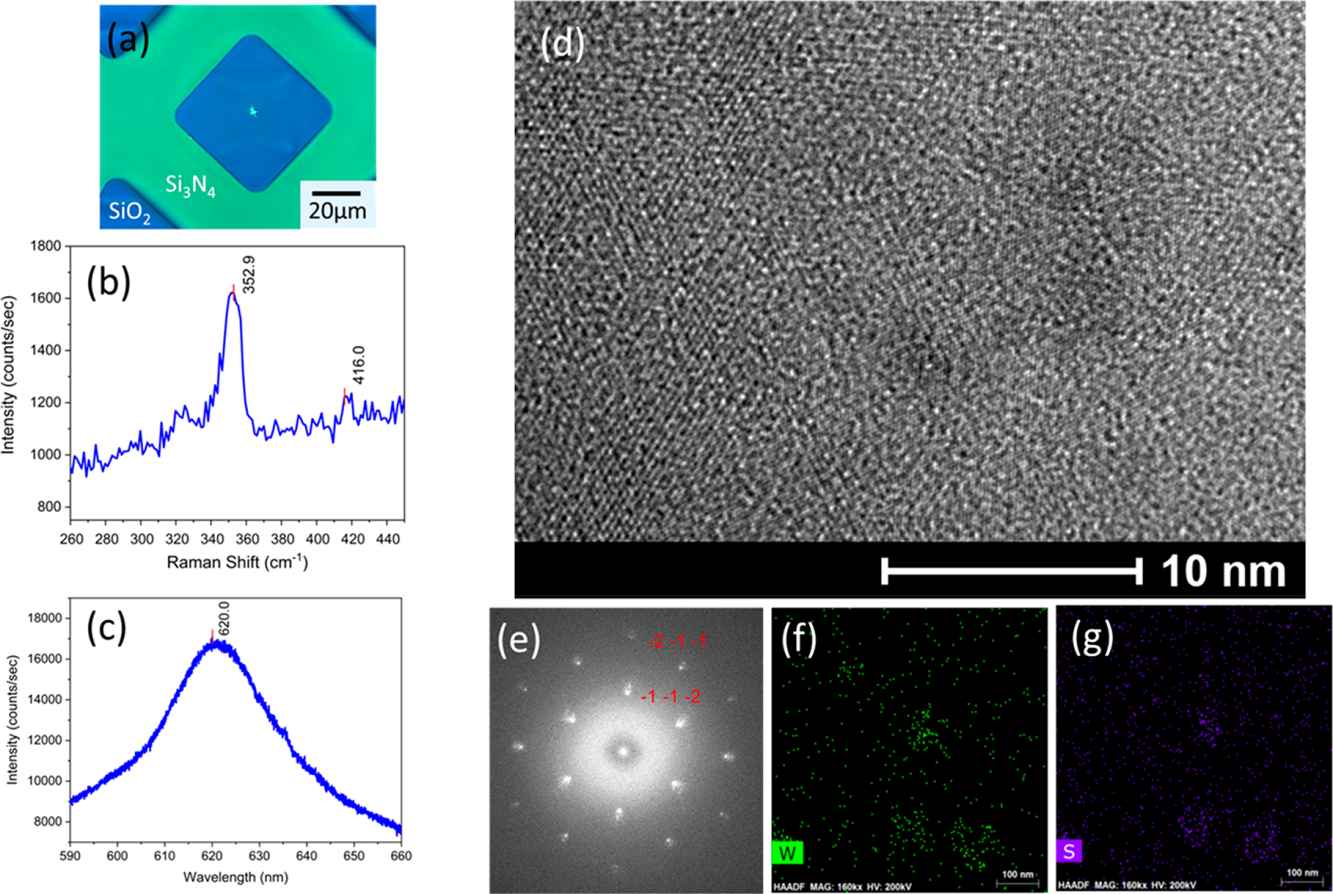
TEM measurements of the VdWE-grown WS_2_ monolayer on a 40 nm SiO_2_ membrane/Si_3_N_4_/Si TEM grid: (a) photograph of the sample with a 532 nm laser spot, (b) Raman spectrum of the VdWE-grown WS_2_ monolayer sample, (c) PL spectrum of the VdWE-grown WS_2_ monolayer sample, (d) TEM image of the VdWE-grown MoS_2_ monolayer sample, (e) SAED patterns of the VdWE-grown WS_2_ monolayer sample, (f) energy-dispersive X-ray spectroscopy (EDX) mapping of Mo atom on the selected area of WS_2_ monolayer sample, and (g) EDX mapping of S atom on the selected area of the WS_2_ monolayer sample.

The optical image of as-deposited WS_2_ monolayer on TEM grid is shown in Figure 6a with a 532 nm laser spot on the center of the WS_2_/40 nm SiO_2_ membrane. The Raman spectrum of the WS_2_ monolayer/40 nm SiO_2_ sample is shown in Figure 6b. Two WS_2_ Raman peaks—2LA phonon mode and A_1g_ mode—were found at 352.9 and 416.0 cm^–1^, respectively. In addition, as shown in Figure 6c, the direct band emission at 620.0 nm (2.00 eV) was revealed from the PL spectrum. Again, these results agree with the literature reports.^26,34^ The TEM image shown in Figure 6d has revealed the polycrystalline nature of VdWE-grown WS_2_ monolayer on 40 nm SiO_2_ TEM membrane, and the grain sizes are ∼10 nm. The SAED pattern shown in Figure 6e also confirmed the polycrystalline structures of this WS_2_ monolayer. The elemental mapping was performed in the STEM-EDX mode. As shown in Figures 6f and 6g, the W and S atoms, respectively, were quite uniform over the measured area.

**Figure 7:**
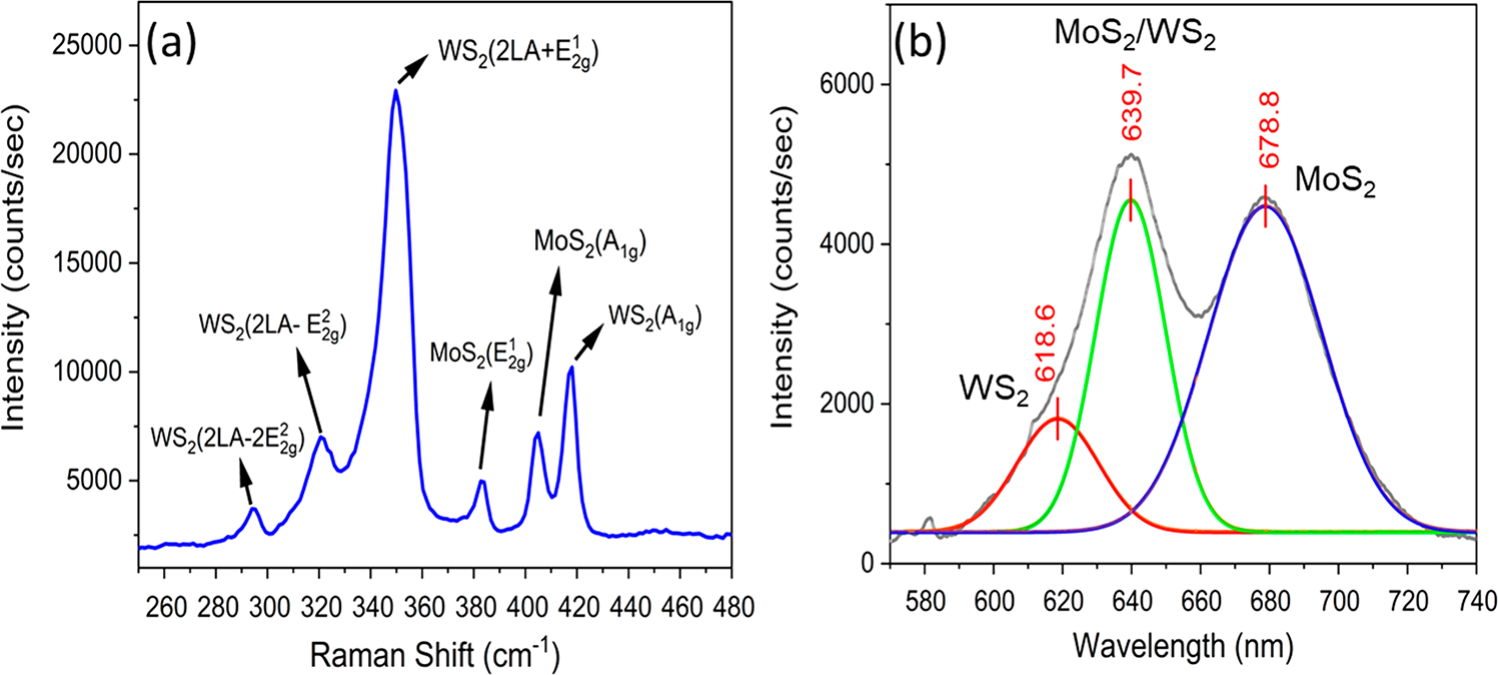
(a) Raman spectrum of MoS_2_/WS_2_ heterostructure on fused silica. (b) PL spectrum of MoS_2_/WS_2_ heterostructure on fused silica.

A MoS_2_/WS_2_ monolayer heterostructure on the fused silica substrate was prepared for further investigation with the above-mentioned VdWE process. WS_2_ monolayer was first grown on a 25 mm × 25 mm fused silica substrate, followed by the second MoS_2_ monolayer grown on the top of a WS_2_ monolayer/fused silica sample. As the Raman spectrum shown in Figure 7a, two typical MoS_2_ E_2g_^1^ and A_1g_ peaks are revealed, along with the WS_2_ peaks labeled as WS_2_(2LA-2E_2g_^2^), WS_2_(2LA-E_2g_^2^), WS_2_(2LA+E_2g_^2^), and WS_2_(A_1g_). The band alignment of MoS_2_/WS_2_ monolayer heterostructures has also been evaluated with the PL spectrum shown in Figure 7b, which revealed that the VdWE-grown MoS_2_/WS_2_ on the fused silica sample forms a type-II heterojunction (more detailed discussion is given in the Supporting Information).

It is very difficult to see the contrast between MoS_2_ and WS_2_ monolayers in the VdWE-grown MoS_2_/WS_2_ heterostructures, since the VdWE provides uniform and continuous atomically thin TMDCs. To visualize the MoS_2_/WS_2_ heterostructures, MoS_2_ monolayer flakes were prepared on a 300 nm SiO_2_/Si substrate with the conventional CVD process,^38^ followed by the coating with a uniform WS_2_ monolayer with the VdWE process. The structure of these VdWE-grown WS_2_ continuous film/CVD-grown MoS_2_ flakes heterostructures illustrated in Figure S1(a) in the Supporting Information with the optical image in Figure S1(b) in the Supporting Information. The detailed characterizations of AFM, Raman, XPS, and PL are discussed in the Supporting Information (Figure S1).

**Figure 8:**
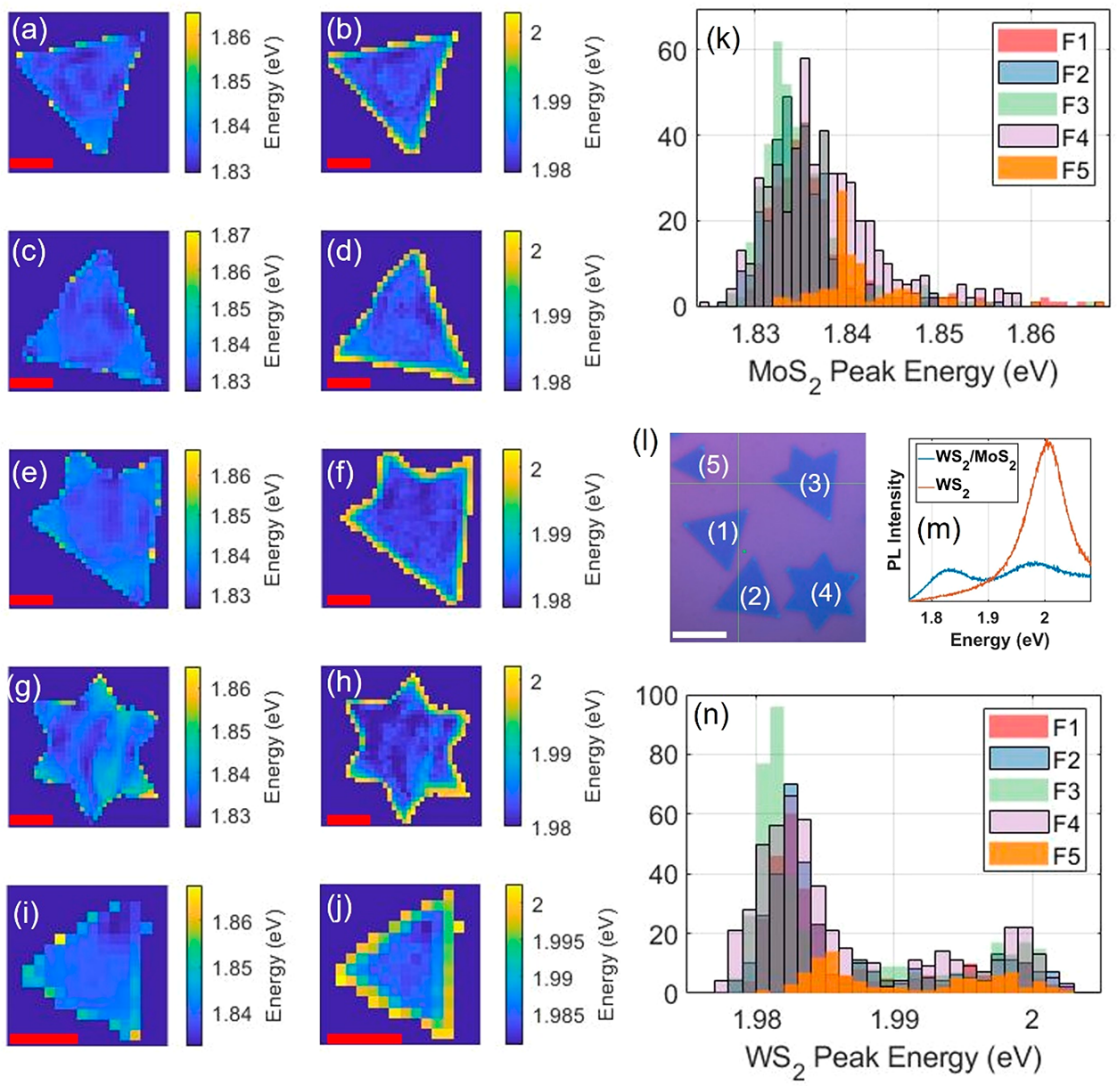
Intraflake and interflake PL spatial and spectral uniformity analysis for five different flakes, as indicated by the optical image (l) with a white scale bar representing 10 μm, on the sample with VdWE-grown WS_2_ monolayer on CVD-grown MoS_2_ monolayer flakes heterostructure on 300 nm SiO_2_/Si. (a, c, e, g, i) Maps of fitted MoS_2_ peak energies for flakes F1–F5, with red scale bars representing 5 μm. (b, d, f, h, j) Maps of fitted WS_2_ peak energies for flakes F1–F5, with red scale bars representing 5 μm. (k) MoS_2_ and (n) WS_2_ show peak energy histograms for flakes F1–F5, plotted on top of each other; all histograms have energy bins 1 meV wide. Panel (m) shows a typical PL spectrum measured from the heterostructure and the surrounding WS_2_.

The spatial uniformity in the VdWE WS_2_/MoS_2_ heterostructures are investigated by PL mapping. A recent report^39^ has shown that the PL uniformity in exfoliated 2D materials is strongly correlated to the uniformity in the spectral properties, such as the emission energy and spectral weighting. A similar analysis is applied here to investigate the uniformity of the heterostructures, in terms of the emission energies of each of the corresponding layers in the heterostructure and offer a baseline for comparisons with future studies. Since there is an abundance of heterostructures flakes, the uniformity analysis extends naturally from intraflake (within one heterostructure flake) to interflake (between multiple flakes), which could provide additional insight for future growth optimizations. The monolayer MoS_2_ flakes on this sample are mostly equilateral triangles, hexagrams, and partial hexagrams of various sizes and orientations. To sample this geometric distribution, an area is selected using optical microscopy, shown in Figure 8l that contains five numerically labeled flakes: flakes F1, F2, and F5 are triangles, F4 is a hexagram, F3 is a partial hexagram, and the regions outside of these flakes correspond to the VdWE WS_2_ monolayer film. A PL map of the entire region was acquired, using a Horiba LabRAM spectrometer, with a 532 nm laser (637 kW/cm^2^, 5 s integration time), focused through a 100× 0.95 NA objective lens, and the emission dispersed with a 600 lines/mm grating. The mapped region is 40 μm × 40 μm in size, and the raster scan step size is 0.5 μm. Maps of individual heterostructure flakes were then isolated from the recorded PL map by a MATLAB program. Figure 8m shows that the WS_2_ region has a single peak at ∼2.00 eV (WS_2_ exciton), while two peaks appear in the heterostructure spectrum at ∼1.84 eV (MoS_2_ exciton) and ∼1.98 eV (WS_2_ exciton).

Spatial variations in emission energy are apparent for both MoS_2_ and WS_2_, as revealed from the peak energy maps in Figures 8a–j. Across all flakes, the peak energy from both materials exhibits similar spatial patterns, where a local area that indicate blue-shifts (or red-shift) in one material corresponds to blue-shifts (or red-shift) in the other at the same spatial location. Although, for the MoS_2_ peak, its intraflake energy range, taken as the 95% confidence region in the histograms shown in Figure 8k, is up to ∼10 meV, compared to ∼4 meV for that of WS_2_, from Figure 8n. There is a pronounced edge effect for WS_2_, less so for MoS_2_, where the peak appears to exhibit a significant blue-shift at the edge of all heterostructures measured. This also explains the differences that are apparent from the histograms plotted in Figures 8k and 8n, showing largely monomodal distribution for MoS_2_ and bimodal for WS_2_. The two modes in Figure 8n corresponds to the interior and edge peak energy distributions for WS_2_, and the means of these two modes are separated by ∼17 meV. The fact that all measured flakes exhibit similar behavior, independent of the flake size, geometry, and orientation, suggests that strain is the likely mechanism to explain this, as its magnitude could be changed at the edge WS_2_ layer as its substrate changes from MoS_2_ to silicon dioxide. For MoS_2_, the peak shift at the flake-edge is much less pronounced, up to 5 meV on average, which is smaller than the inhomogeneity in the MoS_2_ peak energy of ∼10 meV, so that this modal separation is apparent only in the smallest flake measured (F5). Overall, the interflake uniformity is well-behaved, i.e., does not fluctuate significantly from flake to flake regardless of size geometry and orientation, which suggests that the growth process has good reproducibility between heterostructures. The intraflake uniformity is also well-behaved if the edge effects can be ignored, which could be valid for large-area heterostructure flakes. However, charge transport phenomena at the edge that change this behavior, which could be an interesting avenue to explore in a future study with a device, because PL-uniformity analysis alludes to the optical transport phenomena only.

## 4 Conclusion

In conclusion, we have demonstrated a scalable fabrication process for TMDC monolayers and their heterostructures by van der Waals epitaxy. These VdWE-grown MoS_2_, WS_2_ monolayers, and their heterostructures have been successfully deposited on CMOS-compatible substrates, such as 300 nm SiO_2_/Si wafers, quartz glass, fused silica, and sapphire. Detailed characterizations of these TMDCs materials have been performed with SEM, AFM, XPS, micro-Raman, micro-PL, TEM, EDX, and SAED techniques and the band alignment and large-scale uniformity of MoS_2_/WS_2_ heterostructures has also been evaluated with spatially resolved PL spectroscopy. These results have demonstrated not only the excellent characteristics of MoS_2_ and WS_2_ monolayers with large-scale uniformity but also the feasibility of large-scale TMDCs heterostructures that can be achieved by the VdWE in this work. We believe this process and resulting large-scale MoS_2_, WS_2_ monolayers and their heterostructures have demonstrated promising solutions for the applications in next-generation nanoelectronics, nanophotonics, and quantum technology.

## Supporting Information Available

Additional band structure calculations of VdWE-grown MoS_2_/WS_2_ on fused silica sample and detailed characterization of VdWE-grown WS_2_/CVD-grown MoS_2_ heterostructures on a 300 nm SiO_2_/Si substrate (PDF)
