# Supplementary material for: Facilitating Uniform Large-Scale MoS2, WS2 Monolayers, and Their Heterostructures through van der Waals Epitaxy
Source: ACS Appl Mater Interfaces. 2022 Sep 9;14(37):42365–73. doi: 10.1021/acsami.2c12174 (PMC9501908; doi:10.1021/acsami.2c12174)
Supplement: Supplementary file 1 — am2c12174_si_001.pdf [file am2c12174_si_001.pdf]

Supporting Information

Facilitating Uniform Large-scale MoS_2, WS_2 Monolayers and Their Heterostructures Through Van der Waals Epitaxy

Chung-Che Huang[*1], He Wang[2], Yameng Cao[3], Ed Weatherby[1], Filipe Richheimer[3], Sebastian Wood[3], Shan Jiang[4], Daqing Wei[4], Yongkang Dong[5], Xiaosong Lu[6], Pengfei Wang[7], Tomas Polcar[2] and Daniel W. Hewak[1]*

[1]Chung-Che Huang, Ed Weatherby, Daniel W. Hewak, Optoelectronics Research Centre, University of Southampton, Southampton, SO17 1BJ, United Kingdom

[2]He Wang, Tomas Polcar, nCAT, University of Southampton, SO17 1BJ, United Kingdom

[3]Yameng Cao, Filipe Richheimer, Sebastian Wood, National Physical Laboratory, Teddington, TW11 0LW, United Kingdom

[4]Shan Jiang, Daqing Wei, School of Materials Science and Engineering, Harbin Institute of Technology, 150001 Harbin, China.

[5]Yongkang Dong, National Key Laboratory of Science and Technology on Tunable Laser, Harbin Institute of Technology, 150001 Harbin, China.

[6]Xiaosong Lu, School of Physics and Electronic Engineering, Jiangsu Normal University, 221116 Xuzhou, China

[7]Pengfei Wang, Key Laboratory of In-fiber Integrated Optics of Ministry of Education, College of Science, Harbin Engineering University, 150001 Harbin, China

*Corresponding author: cch@orc.soton.ac.uk

In this section, we provide additional band structure calculations of VdWE-grown MoS_2/WS_2 on fused silica sample. This particular junction[1] shows MoS_2 (band gap 2.39 eV) and WS_2 (band gap 2.31 eV) form a staggered junction where the difference between valence band maxima is around 0.35 eV. Excitons (X_0) form in MoS_2 and WS_2 with binding energies of 0.53 eV and 0.3 eV respectively, leading to emissive photoluminescence bands with peak energy 1.86 eV (666.3 nm) and 2.01 eV (616.1 nm) respectively. MoS_2 can form charged exciton (CX) states, with a binding energy of ~ 0.03 eV and emits at 1.83 eV (678.8nm). Due to the staggered band alignment, holes from MoS_2 can relax into the valence band of WS_2 and the electrons from WS_2 can relax

into the conduction band of MoS_2, leading to indirect excitons (IX) at 1.94 eV (639.7 nm) with electron and hole spatially separated in the heterostructures. These results are in very good agreement with the literature.[2]

Moreover, we provide additional characterization of VdWE-grown WS_2/CVD-grown MoS_2 heterostructures. Figure S1(a) illustrates the cross-section structure and S1(b) shows the optical image of VdWE-grown WS_2/CVD-grown MoS_2 heterostructures deposited on SiO_2/Si substrate. Although the triangular flakes can be seen, their nature needs to be characterized further. According to the surface topographic image shown in Figure S1(c) scanned by tapping mode AFM, the step height of VdWE-grown WS_2/CVD-grown MoS_2 flake heterostructure on SiO_2/Si substrate (after removal of the monolayer WS_2 on SiO_2/Si substrate by the AFM tip) marked in Figure S1(b) is ~1.62 nm, indicating the thickness of hetero-bilayer WS_2/MoS_2 heterostructures. As evident from Figure S1(d) and S1(e), the overall Raman and PL signals of the WS_2/MoS_2 heterostructures are independently contributed by MoS_2 (383.2 and 404.2 cm^{-1}, frequency difference is 21.0 cm^{-1}) and WS_2 (350.2 and 419.9 cm^{-1}, frequency difference is 69.7 cm^{-1}). Compared with the frequency differences between E^1_{2g} and A_{1g} modes for single-layer MoS_2 (20.6 cm^{-1})[3] and WS_2 (67.2 cm^{-1})[4] using 532nm excitation laser, these values are larger in the case of a heterostructure, which can be

ascribed to the stacked bilayer structure. A similar phenomenon can be noticed from the PL spectrum: the emission peaks for WS_2/MoS_2 heterostructure are shifted to the higher wavelength end in contrast to MoS_2 (676.0 nm) and WS_2 (620.2 nm) monolayer. An XPS scan was performed for the WS_2/MoS_2 heterostructure to confirm its elemental compositions. As displayed in Figure S1(f)-S1(i), the peaks at 226.4, 229.1, 232.3, 32.3, 34.5, 162.1, 163.2 eV correspond to the S 2s, Mo $3d_{5/2}$, Mo $3d_{3/2}$, W $4f_{7/2}$, W $4f_{5/2}$, S $2p_{3/2}$ and S $2p_{1/2}$ orbits, and the carbon 1s peak at 284.7 eV indicates that the pure C-C bonding is revealed and there is no significant chemisorption of carbon.

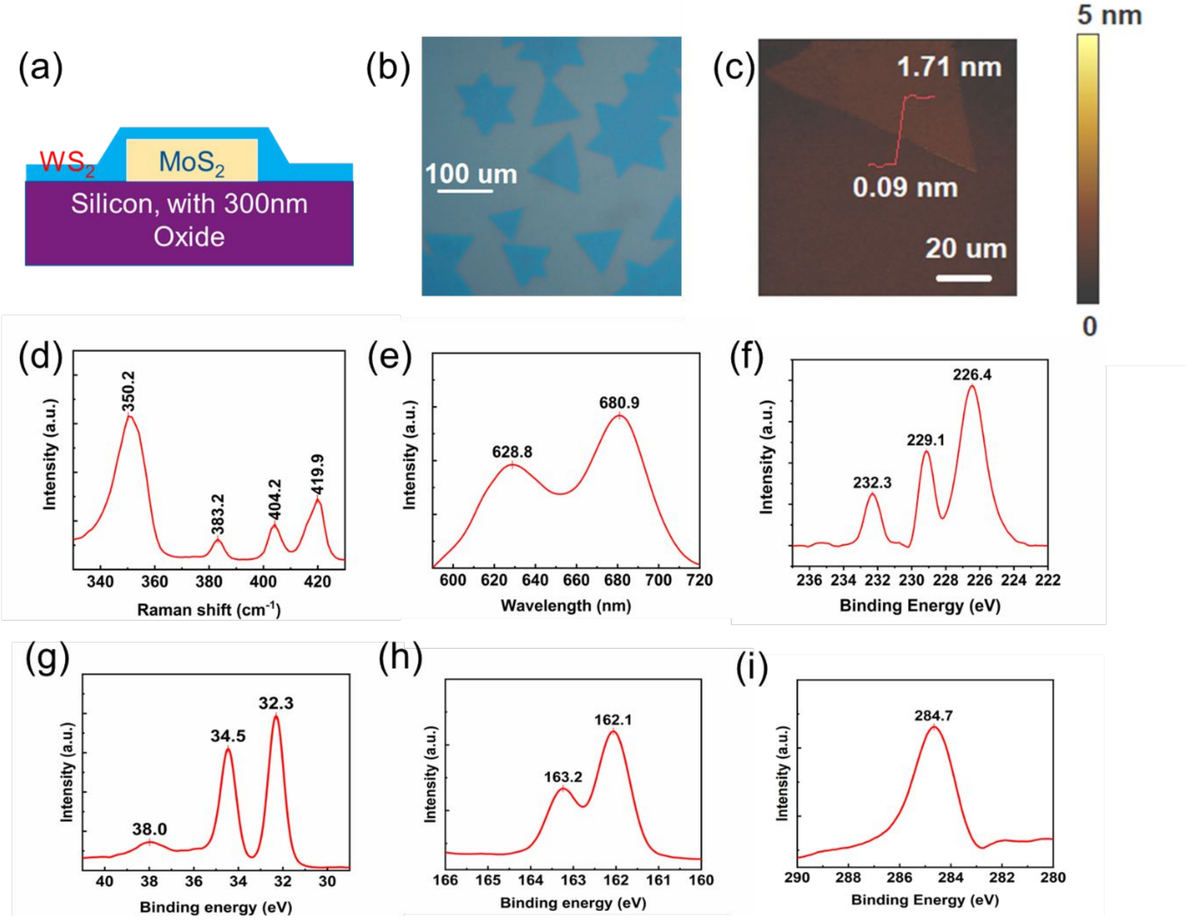

Figure S1 (a) illustration of sample cross-section structure of VdWE-grown WS_2 monolayer on CVD-grown MoS_2 monolayer flakes heterostructure on 300 nm SiO_2/Si (b) Image of VdWE-grown WS_2 monolayer on CVD-grown MoS_2 monolayer flakes heterostructure on 300 nm SiO_2/Si (c) AFM measurement of VdWE-grown WS_2 monolayer on CVD-grown MoS_2 monolayer flakes heterostructure on 300 nm SiO_2/Si (d) Raman spectrum of VdWE-grown WS_2 monolayer on CVD-grown MoS_2 monolayer flakes heterostructure on 300 nm SiO_2/Si (e) PL spectrum of VdWE-

grown WS_2 and VdWE-grown WS_2/CVD-grown MoS_2 monolayer flakes on 300 nm SiO_2/Si (f) XPS analysis of Mo 3d scan (g) W 4f scan (h) S 2p scan and (i) C 1s scan of VdWE-grown WS_2 monolayer on CVD-grown MoS_2 monolayer flakes heterostructure on 300 nm SiO_2/Si

REFERENCES

(1) Koåmider, K.; Fernández-Rossier, J. Electronic Properties of the MoS2-WS2 Heterojunction. *Physical Review B - Condensed Matter and Materials Physics* **2013**, *87* (7). https://doi.org/10.1103/PhysRevB.87.075451.

(2) Zhang, J.; Wang, J.; Chen, P.; Sun, Y.; Wu, S.; Jia, Z.; Lu, X.; Yu, H.; Chen, W.; Zhu, J.; Xie, G.; Yang, R.; Shi, D.; Xu, X.; Xiang, J.; Liu, K.; Zhang, G. Observation of Strong Interlayer Coupling in MoS2/WS2 Heterostructures. *Adv Mater* **2016**, *28* (10), 1950–1956. https://doi.org/10.1002/adma.201504631.

(3) Wang, H.; Huang, C. C.; Polcar, T. Controllable Tunneling Triboelectrification of Two-Dimensional Chemical Vapor Deposited MoS2. *Scientific Reports* **2019**, *9* (1). https://doi.org/10.1038/s41598-018-36830-1.

(4) Wang, H.; Huang, C. C.; Polcar, T. Triboelectrification of Two-Dimensional Chemical Vapor Deposited WS2 at Nanoscale. *Scientific Reports* **2019**, *9* (1). https://doi.org/10.1038/s41598-019-49107-y.
